# Multi-Agents-Based Modeling and Simulation for Carbon Permits Trading in China: A Regional Development Perspective

**DOI:** 10.3390/ijerph17010301

**Published:** 2020-01-01

**Authors:** Junjun Zheng, Mingmiao Yang, Gang Ma, Qian Xu, Yujie He

**Affiliations:** School of Economic and Management, Wuhan University, Wuhan 430072, China; zhengjunjun@whu.edu.cn (J.Z.); mingmiao.yang@whu.edu.cn (M.Y.); 2017201050237@whu.edu.cn (Q.X.); 2016302350027@whu.edu.cn (Y.H.)

**Keywords:** multi-agents, carbon permits trading, simulation analysis, regional development

## Abstract

China has been actively taking actions to control carbon emissions and promoting development of a carbon market. However, there are many disadvantages in a carbon market, owing to various designs and policies still under trial and implementation. Adopting the multi-agents technique, we constructed a framework about national carbon market to estimate the effect of a different design of policy made on the GDP (Gross Domestic Product) and environment. In particular, national and regional abatement policies were analyzed in our study. The results showed the carbon-trading mechanism can effectively reduce carbon emissions and make a negative impact on GDP. National abatement can neither be too high nor too low for reducing carbon emissions and maintaining economic stability. For different regions, the central region was impacted the most by a carbon trading mechanism, and the east region was the opposite. Moreover, the “sweeping approach” policy should be replaced by a regional “discriminating policy” because the abatement requirement to the western region was low and to the eastern region was relatively high, which is more beneficial to China’s regional development.

## 1. Introduction

China, which is regarded as a developing country, has been recognized as becoming the largest carbon emitter in the world with rapidly increasing CO_2_ emissions [1]. It became the world’s largest carbon emitter in 2006, and was responsible for 64.8% of the increase in global carbon emissions between 2007 and 2012. Moreover, in 2013, carbon emissions of China approximately made up 28.6% of the world’s total carbon emissions [2,3]. As China is facing great pressure from the international community on the issue of carbon emissions, the government is making an effort to control the carbon emissions. In 2014, China pledged, in the US–China Joint Announcement on Climate Change, that it would reach its peak of carbon emissions around 2030 [4]. It is noticed that climate change has worsened the economy. Like the Stanford study showed, China (population 1.4 billion) has seen −1.4% of median change in per capita GDP (Gross Domestic Product) from global warming between 1961 and 2010 [5,6]. If future adaptation mimics past adaptation, unmitigated warming is expected to reshape the global economy by reducing average global incomes roughly 23% by 2100 [7].

This has put China’s government in an alarming condition to reform this problem by actively taking responsibility for carbon emission abatement and also to control greenhouse gas in the overall strategy of society development. Having realized the importance of how carbon emissions directly affect the economic growth status, China has mainly taken measures of decreasing emission intensity to control the total emissions, including abatement targets: Carbon emissions of per unit GDP in 2020 should be 40%–45% lower than 2005, presented in the 13th five plan [8], building carbon-trading pilots nationally and other policies. Before October 2018, eight pilot projects were built, namely Shenzhen (6, 2013), Shanghai (11, 2013), Beijing (11, 2013), Guangdong (12, 2013), Tianjin (12, 2013), Hubei (4, 2014), Chongqing (6, 2014), and Fujian (12, 2016). Moreover, in order to further promote green development, the National Development and Reform Commission (NDRC) issued the national carbon emission trading market construction plan (power generation industry) in December 2017, marking the completion of the overall design and formal launch of China’s carbon-trading system. A pollution control policy has proven to be effective in some economically developed regions of China [9].

Moreover, the proposed regulatory system has a huge impact on the public. It is reflected not only in stabilizing the economy to make sustainable development practical, but also in environmental improvement for the well-being of future generations. When many pollutants, led by carbon emissions, are effectively controlled, public health can be guaranteed.

Carbon emission reduction has become an important issue in today’s world. However, the balance of regional economic development needs to be emphasized, where eastern regions have denser populations and a more advanced economy than western regions. If it aims for the same target, the western regions, with a high consumption of coal resources and relatively rich, heavy chemical industries that highly consume energy and produce lots of pollution, will confront negative effects on the economy and development. Therefore, from the perspective of the regional development, this paper has an important practical significance to indirectly explore the effects of carbon emissions.

The remainder of this paper is organized as follows: Section 2 is the literature review. Section 3 lays out a model framework of how these agents make decisions and shows data we used. Section 4 depicts the simulation results and comparisons. Section 5 compares the present results with that from previous studies and discusses the abatement policy then gives some policy implication. Section 6 provides some concluding remarks and research extensions.

## 2. Literature Review

In the development of China’s carbon market, many potential challenges emerge [10]. As an effective abatement tool, much literature has studied how the mechanism performs and the impact brought about, like the allocation of free quotas [11,12] and trading activities [13,14,15]. The impact brought about by carbon-trading mechanism is negative on GDP [16].

Notably, the aforementioned studies were performed in light of macro-scale analysis. In this paper we used multi-agents technology as an analysis method. Many previous policy simulation analyses focused on typically top-down methods [17], like computable general equilibrium (CGE) [18] and DEA(Data Envelopment Analysis) [19,20], which cannot reflect realistic trade, individual behavior or process of decision. Therefore, adopting the bottom-up simulation model that fully considers agent behaviors and effect to the whole system is significant [21]. One of the most common models is the bottom-up multi-agents model, which can appropriately describe the effect each agent’s behavior has on carbon-trading based on the micro-individual view. It has some characteristics: (1) Heterogeneity is when the individual agent is depicted with characteristics and attributes [22]. (2) Adaptivity is when the external environment changes, different agents conduct different adjustment behaviors to better adapt themselves to these changes of environment [23]. (3) Learnability is when each agent is rational, but the learning ability is different. Using learning ability, agents can choose a strategy of maximum benefit through the adjustment of historical experience and their own behavior [24]. Wu et al. [25] used a multi-agents model to analyze the influence of enterprise innovation on energy consumption and found that carbon emissions and energy consumption will reach the highest point in 2027 and 2028, respectively. Natara et al. [26], from the residents’ point of view, studied the characteristics of carbon emission. Cong et al. [27] just paid attention to the mutual effect between policy and power sector, except impact policy made on other sectors. Tang et al. [16,28] made analysis on nationwide policy simulations of the impact on GDP and the environment but without considering regional differences.

In addition, most existing papers just focused on the national carbon market [16,29], certain sectors [30,31,32], or certain regions [33,34], ignoring the regional imbalance. When it comes to abatement policy, the balance of regional economic development needs to be emphasized, where eastern regions have denser populations and a more advanced economy than western regions. Various regions are characterized differently in both economy and emission [35,36], especially in features of carbon emissions reduction capacity, responsibility, potential, and energy efficiency [37,38]. Therefore, it is not practical for compulsive requirement of each province to set the same mitigation target owing to differentiated specific native industrial structure. In other words, different provinces or regions are supposed to share different mitigation emission standards. In pursuit of effectively controlling emissions and protecting native economic development, the government should make carbon policy from a regional perspective [39,40,41].

To solve these problems it is important to consider two aspects: One is considering the bottom-up agents interacting, the other one is exploring the carbon-trading market from the regional perspective. Our paper adopted the multi-agents model but simulates China’s carbon trading from a regional perspective, which is to illustrate what effect the different policies can bring to both GDP and environment. Explicitly, we adopted the multi-agents model to capture the sectors’ activities and interactions among various heterogeneous agents under the carbon trading rather than CGE to explore the suitable carbon-trading design in China, in which different abatement scenarios were designed and analyzed. Moreover, taking regional economic development differences into account in our model, we divided China into 3 regions: Eastern, central, and western. Then we explored how the effect different policies made on GDP and the environment through simulation processes which included totally 900 sectors agents covering 30 cities.

Therefore, our work contributes two aspects as follows: (1) This paper adopted a multi-agents model approach through Chinese multi-regional input-output (MRIO) data for 2012 [42], which is a bottom-up technology. Different from most previous literature that analyzed policy simulation typically using top-down methods, we used a multi-agents model to explore an appropriate abatement policy. Moreover, MRIO data for 2012 covered 900 sectors agents covering 30 cities, which was the latest data many papers could use. (2) According to different levels of regional economic development, a carbon-trading market from the regional perspective was studied in our work. Owing to the characteristic of various regions being reflected differently in both economy and emission, it was vital for a discriminating requirement of each province to set a mitigation target. We divided China into three regions: Eastern, central, and western, respectively. Furthermore, we designed six different abatement policies, M1–M6, to compare a “discriminating policy” that sets different abatement targets for regions with a “sweeping approach” that sets the same abatement target on each province. In general, this study set the target to build a nationwide China carbon-trading simulation model by multi-agents technology, where the regional development demands especially analyzed for China’s economy and abatement.

## 3. Materials and Methods

### 3.1. Model Framework

**Figure 1:**
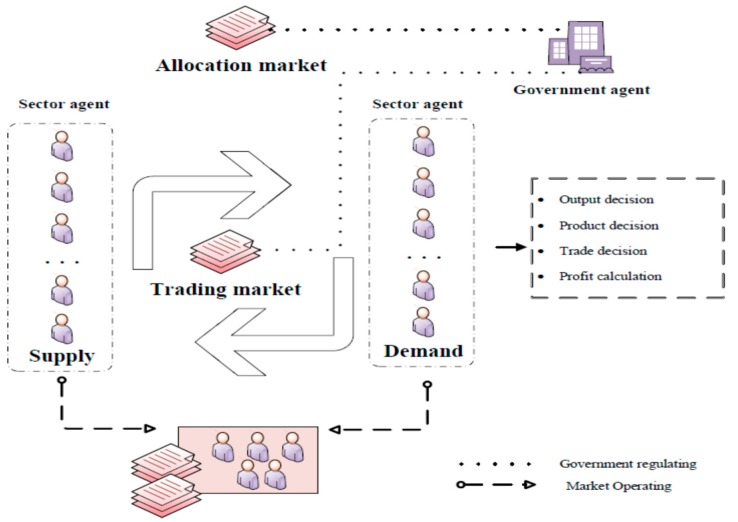
Framework of agents and markets.

In the model we set up, two main types of agents are described: Government and sectors. The interaction between both kinds of agents through two carbon markets, including the allocation market and trading market, is depicted by framework (Figure 1).

#### 3.1.1. Agents’ Description

We show two kinds of agents. One is the government agent, the other one is the sector agent.

1. The government, as the carbon-trading designer and supervisor, has the following behaviors: (1) It calculates the individual and total carbon emissions, (2) allocates free carbon emission quotas to sectors, (3) imposes penalties on unwarrantable emissions (i.e., excess emission beyond the owned allowances), (4) regulates the trading price, and (5) conducts its economic activities (levying taxes, consuming commodities, and saving).
2. The sectors, as the carbon-trading targeted agents, make optimal decisions for production scheme, obtain the freed allocated allowances (in the allocation market), make transactions of carbon emissions (in the trading market), and sell commodities.

#### 3.1.2. Markets’ Description

China’s carbon-trading market is divided into two sections, the allocation market and trading market, respectively. Quotas allocated in the allocation market are mainly based on the free allocation pattern, which is derived from demanding accordance with stage characteristics of China’s low carbon transition. The allocating methods for free quotas are various, in the light of different rules such as historical emissions, all sector emissions, future emissions, and so on [43]. In this paper, we used the popular method: Grandfathering rule. After allocation, each participator makes a trading strategy according to their own actual emissions in the trading market, which promotes the trade of quotas or carbon emission permits [16]. Shenzhen is taken as an example of the pioneer in carbon-trading projects, which is the first pilot project to implement cap-and-trade [43,44] and can accelerate reforms of reducing emissions and accomplishment of abatement by gradually lowering the emission cap of participators in the carbon-trading system [45]. Additionally, cap-and-trade was adopted in this paper to conduct a scenario simulation analysis. Furthermore, the trading market is a buyer’s market when demand is more than supply; otherwise it is a seller’s market. According to adjustment of the market, the market price of carbon fluctuates over time.

### 3.2. Government Agent

The government has two main purposes: To control the carbon emissions and to stimulate GDP. Therefore, how to balance both aims is our focus to explore. Facing the carbon emission market, the government needs to take some measures to control it.

#### 3.2.1. Calculation of Carbon Emissions

The government has to strictly calculate individual carbon emissions by considering the fossil energy during production [46]:

(1) $$Qemission_{i,j,t}=\sum_{n=1}^{4}a_{n}b_{n}c_{n}F_{n,i,j,t}\times \frac{44}{12},$$

where *Qemission_i,j,t_* is the carbon emission from sector *j* in province *i* at time *t*, and *F_n,i,j,t_*, *n* = 1, 2, 3, 4 denote the fossil energy n in production including coal mining (COM), petroleum and gas (PEG), petroleum refining, coking, etc., (PRC) and gas and water production and supply (GWP). Additionally, *a_n_*, *b_n_*, and *c_n_* are the conversion factor, emission factor, and oxidization factor of fossil energy *n*, respectively.

#### 3.2.2. Allocation Rule

In terms of reducing total carbon emissions, the paper considered the prevalent grandfather rule that is based on the historical carbon emissions of the individual sector.

For the grandfathering policy, the government uses emissions reduction factor $\eta_{i,j,t}$ to control the free carbon allowances supply in the carbon-trading market. This factor in our study was treated differently according to eastern, central, and western regions, which is detailed in the subsection of Setting of Different Scenarios. Therefore, carbon emissions of sector *j* in province *i* at time *t*, *Qallpwance_i,j,t_* is concluded as Equation (2).

(2) $$Qallowance_{i,j,t}=(1-\eta_{i,j,t})Qemission_{i,j,t-1},$$

#### 3.2.3. Cap-and-Trade

If individual actual carbon emissions are in excess of a carbon permits quantity, i.e., $Qemission_{i,j,t}>Qpermit_{i,j,t}$, the sector *j* in province *i* should face the penalty, $Penalty_{i,j,t}$, at time *t*.

The carbon permits, *Qpermit_i,j,t_*, include free allowance and trading from the carbon emissions trading market, $Qpermit_{i,j,t}=Qallowance_{i,j,t}+Qtrading_{i,j,t}$. When *Qtrading_i,j,t_* is non-negative, it means that the sector *j* in province *i* buys quotas from others who have more quotas than they need.

Because the carbon allowance is constrained by the government to effectively control the carbon emissions, some individual sectors will encounter a shortage of carbon permits in spite of buying quotas in the trading market.

As a result, the illegal emission is derived as $Iemission_{i,j,t}=Qemission_{i,j,t}-Qpermit_{i,j,t}$, the penalty price is $pd_{t}=\phi pt_{t}$, where ϕ is a penalty multiplier and *pt_t_* is the trading price in the trading market.

(3) $$penalty_{i,j,t}=\{\begin{array}{c} 0,Iemission_{i,j,t}<0\\ pd_{t}\times Iemission_{i,j,t},Iemission_{i,j,t}\leq Cap/pt\\ Cap,Iemission_{i,j,t}>Cap/pt \end{array},$$

where *Cap* is the penalty cap. It is because the carbon-trading market is not yet completed to nationally operate that the marginal cost of exceeding the cap becomes zero. Although the government implements this policy for easing sectors’ pressure to reduce emissions, the penalty cap is frequently used to make many sectors’ penalty the penalty cap.

#### 3.2.4. Economic Activities

First, the government levies various taxes as fiscal revenue *T_t_* and imposes a penalty for illegal emissions, *penalty_i,j,t_*. Second, the government consumes lots of commodities *G_i,j,t_*

(4) $$Saving_{t}=T_{t}+\sum_{i}\sum_{j}penalty_{i,j,t}-\sum_{i}\sum_{j}G_{i,j,t},$$

### 3.3. Sector Agent

In the carbon market, there are two parts, the allocation market and the trading market. Each system participator has to make many decisions during this process, such as output decision, production decision, carbon-trading strategy, and calculation profit.

#### 3.3.1. Output Decision

The sector *j* in province *i* at time t makes the output decision referring to the change of profit. In terms of the learning principle [47], the output strategy *Y_i,j,t,_* is as follows:

(5) $$Y_{i,j,t}=\{\begin{array}{c} Y_{i,j,t-1}+\Delta Y_{t},\pi_{i,j,t-1}-\pi_{i,j,t-2}>\delta \\ Y_{i,j,t-1},|\pi_{i,j,t-1}-\pi_{i,j,t-2}|\leq \delta \\ Y_{i,j,t-1}-\Delta Y_{t},\pi_{i,j,t-1}-\pi_{i,j,t-2}<\delta \end{array},$$

where Δ*Y_t_* and *δ* are non-negative parameters that denote output adjustment and a critical threshold, respectively. When profit increased in the last period, the sectors increased their output level, and the increment is Δ*Y_t_*. For adopting dynamic change, we set $\Delta Y_{t}=0.2Y_{i,j,t-1}$. When the change of profit was in the range of [−δ,δ], the remaining level of output is the decision. When the profit of the last period was decreased, the sector should reduce output.

#### 3.3.2. Production Decision

On the basis of the nested structure to represent different substitutions among various inputs [46], we built a six-layer structure including constant elasticity of substitution (CES) used in five layers and a Cobb–Douglas production function in the sixth layer.

(6) $$Fossil_{i,j,t}={\left(\sum_{n=1}^{4}e_{n}F_{n,i,j,t}{}^{\rho f}\right)}^{1/\rho f},$$

where $\rho f=\left(\sigma_{f}-1\right)/\sigma_{f}$ (*σ_f_* is the elasticity of substitution among four fossil energy) and *e_n_* is the share of energy input.

In the second layer, the energy input $E_{i,j,t}$ is composed by the electricity $ELE_{i,j,t}$ and fossil energy *Foss_i,j,t_*, which is similar to the first layer. The $\sigma_{el}$ is the elasticity of substitution between electricity and fossil energy. In the third layer, energy input $E_{i,j,t}$ and capital $K_{i,j,t}$ construct the energy–capital input $EK_{i,j,t}$, which is then combined into the energy–capital–labor input $EKL_{i,j,t}$ with the labor input $L_{i,j,t}$ in the fifth layer. All of these compositions follow CES technology and are similar to the first layer.

In the fifth layer, the non-energy input, the total intermediate input $Cin_{i,j,t}$ is the sum of intermediate goods from different non-energy input, $Cin_{i,j,t}=\sum_{s}Int_{s,i,j,t}$, where *Ints,i,j,t*, (*S* = 1,2,3,…25) denotes the intermediate input of non-energy commodity *s* used by the sector *j* in province *i* at period *t*. Furthermore, the final output $Y_{i,j,t}$ with a Cobb–Douglas production function is illustrated as follows:

(7) $$Y_{i,j,t}=A\cdot KEL_{i,j,t}{}^{\alpha }Cin_{i,j,t}{}^{1-\alpha },$$

where *A* is a coefficient, and α is the proportion taken by KEL (energy–capital–labor) input.

#### 3.3.3. Carbon-Trading Strategy

Each participator in the carbon market is confronted with balance between permit quotas and actual emission quantities. The trading quantities can be described as Equation (8),

(8) $$Qtrading_{i,j,t}=Qemission_{i,j,t}-Qallowance_{i,j,t},$$

If the free allocation is less than demand, the agents want to buy demand quota in the trading market, $Qtrading_{i,j,t}>0$. Otherwise, the sectors that abound with carbon quota sells the extra emissions, $Qtrading_{i,j,t}<0$.

However, it is obvious that quotas from two trading parts are not always equal, which means some individuals cannot get enough quotas they demand or sell those they want. Therefore, the true trading cost or profit is derived according to the true trading quantity, $\left|qtrading_{i,j,t}|\leq |Qtrading_{i,j,t}\right|$,

(9) $$\mathrm{Cos}tquota_{i,j,t}=pt_{t}\times qtrading_{i,j,t},$$

where *pt_t_* is the trading price of a unit of carbon quota.

#### 3.3.4. Profit Calculation

The sale income of sector *j* in province *i* at time *t*, $Sale_{i,j,t}$ is gained by selling outputs. What they need to pay are various inputs, including energy input ($E_{i,j,t}$), capital investment ($K_{i,j,t}$), labor ($L_{i,j,t}$), intermediate input ($Cin_{i,j,t}$), and tax ($Tax_{i,j,t}$). Additionally, there are some payments in the carbon market, such as the cost for buying needed quotas (Cos*tquota_i,j,t_*) and the possible penalty ($Penalty_{i,j,t}$). The profit $\pi_{i,j,t}$ of sector *j* in province *i* at time *t* is depicted as:

(10) $$\pi_{i,j,t}=Sale_{i,j,t}-E_{i,j,t}-K_{i,j,t}-L_{i,j,t}-Cin_{i,j,t}-Tax_{i,j,t}-\mathrm{Cos}tquota_{i,j,t}-penalty_{i,j,t}.$$

### 3.4. Market

In the allocation market, each sector agent gains the initial carbon allowances, where government allocates the quotas to an individual sector in different provinces according to grandfathering rule, which is shown in Equation (2). Each sector, as a rational agent, has its own output strategy adjustment based on historical profit in Equation (5). Then a relevant input decision is made, in which four fossil energy inputs can bring carbon emissions, which is verified by the government as Equation (1). Subsequently, two situations for verifying results can be described as the demand situation and the supply situation, which means that the sector whose emissions exceed permits needs to buy extra quotas in the trading market, or whose allowances remain so that they truly want to sell redundancies.

As for the trading market, total allowance supply is more than total allowance demand, $Supplyallowances_{t}>Demandallowances_{t}$, which means it is a buyer’s market, or otherwise, the market is a seller’s market. Additionally, for fear of an abnormal price happening in the carbon-trading market, the government regulates the market price $pt_{t}$ by regulating price $pt_{g}$.

(11) $$pt_{t}=\{\begin{array}{c} pt_{g}[1+\frac{Demandallowances_{t}-Supplyallowances_{t}}{Supplyallowances_{t}}],Supplyallowances_{t}>Demandallowances_{t}\\ pt_{g}[1+\frac{Demandallowances_{t}-Supplyallowances_{t}}{Demandallowances_{t}}],Supplyallowances_{t}\leq Demandallowances_{t} \end{array}$$

The price of carbon trading has a signal effect on the individual sector. On the one hand, when the price is high, the signal of the price in the seller’s market helps sectors to make the choice to reduce the production scale to relieve economic stress in the next term. Under this situation, the sector will be apprehensive that both the carbon emission target and the penalty for illegal emissions stimulated by the carbon market directly lead the cost and price of production to increase from the signal. On the other hand, when the price is low, the price in a seller’s market also becomes a signal, in which sectors make different decisions from the signal of high price. As an adaptive learning agent, the sector that is just in pursuit of profits may choose to use more energy input to increase output then gain more profit in spite of penalties for excess emissions. Additionally, the price of a commodity that each producer sells and buys are supposed as exogenous by using a constant value (we use 1) to represent better focus on carbon trading.

### 3.5. Data Description

**Figure 2:**
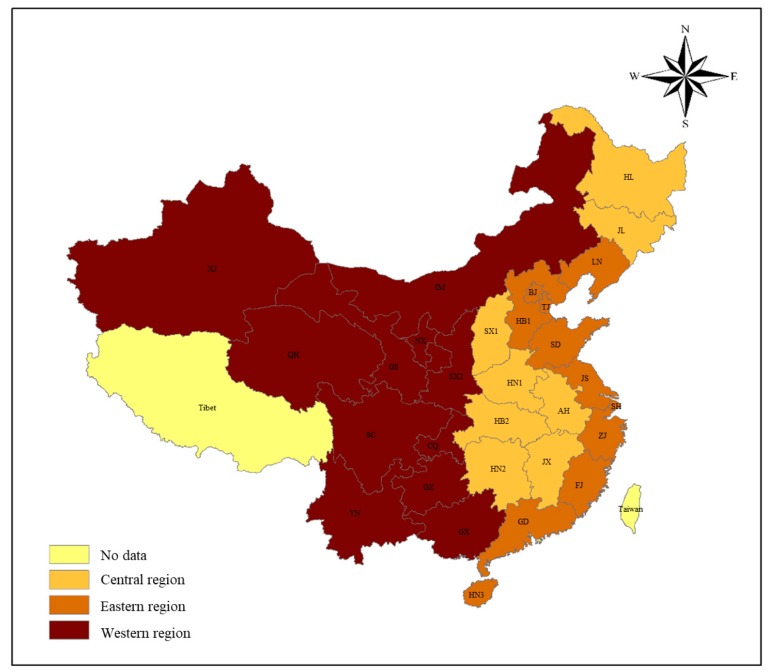
Map of different regions, except the South China Sea islands.

All sectors in China can be classified as 30 sectors, due to different properties of energy consumption and carbon emissions quantities, as showed in Table 1. Four fossil energy sectors are specially researched, owing to their contributions to carbon emissions, but the electricity sector as our focus energy sector does not produce carbon emissions. We mainly introduced 30 counties of China, and divided them into three regions in terms of economic development, which is shown in Table 2 and Figure 2.

For model initialization, we referred to the Chinese multi-regional input-output (MRIO) data for 2012 [42], which provided us data in a base period. Moreover, the source of the data is contained in the appendix of literature [42]. The regional carbon emission was derived from a previous study [21,35,48]. Other initial decisions were assumed as normal distributions [28]. The substitution elasticity parameters were derived from [46]. The factors in carbon emissions were calculated according to the Intergovernmental Panel on Climate Change (IPCC). The emission reduction factor $\eta_{i,j,t}$ was specified according to previous papers [16,49] and the 13th plan [8].

## 4. Results

### 4.1. Setting of Different Scenarios

To explore design mechanisms which affect the abatement, the base case without carbon trading, named as scenario N, is first run, then the typical carbon-trading scenario (T) will follow. In order to maintain the carbon market and avoid speculations, some pilots of China, like Beijing, Shenzhen, and Hubei, would reserve a small amount of allowances to stabilize the carbon-trading price [45]. Therefore, the government controls the price fluctuating around a regulating price that is concluded [24] as 40 yuan metric ton. There is a simple example of scenario N’, in which a carbon emissions increment can bring proportional decrement (0.3) in an economy after normalizing the different unit. Scenario N’ is defined as the situation that effected by climate effect, the impact of no controlling carbon emissions brings to economy. 

As for an abatement policy, during the third phase of the EU (2013–2020), the allowances were expected to decrease by 1.74% per year. Moreover, according to China’s current carbon emissions level and carbon reduction goal, the abatement rate was suggested to be about 3% [16]. Therefore, 1.5%, 3%, and 4.5% were designed into our abatement policies M1–M6.

In conclusion, this paper designed many scenarios (Table 3). Moreover, the calculations for the model were performed up to the 20th year after forcing the carbon market to capture its dynamic impacts on China’s GDP and carbon emissions.

### 4.2. Model Testing Results

To testify to the validity of the proposed model, the provincial GDP and carbon emissions in all sectors of scenario N and real data (MRIO) of the base period were compared, and the simulation errors are listed in Table 4. The absolute errors of China’s total GDP and total carbon emissions of base the period were −0.66% and 0.23%, respectively.

**Figure 3:**
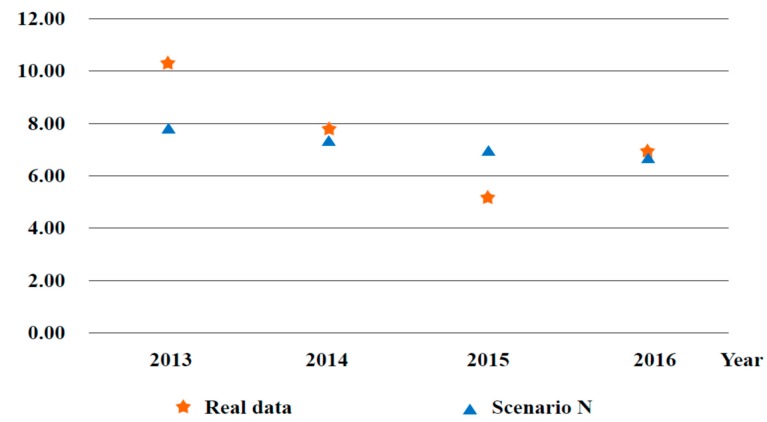
Comparison between real data and simulation data of scenario N in regards to GDP growth rate (shown as percentages).

Furthermore, to check whether the dynamic GDP satisfied the actual situation in the scenario N compared to real data from 2012 to 2017, for explicit description, we used chronological simulation data of growth rate under scenario N compared with real data from the Chinese Statistical Yearbook 2012–2016 and discounted them into 2012, which is shown in Figure 3.

### 4.3. Different Scenarios for Carbon Emissions and GDP

Based on the multi-agents model for policy design, the impacts of carbon trading on China were evaluated by comparing scenario N and scenario T, as shown in a previous section. Then, a series of exploration policies, listed in Table 3, were simulated and are further analyzed in the next section.

**Figure 4:**
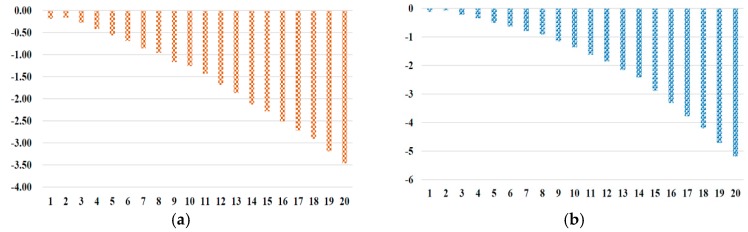
Impacts of the trading mechanism under scenario T (%), (**a**) carbon emissions (**b**) GDP.

As for impacts on carbon emissions and GDP, Figure 4a,b represent details, which are the comparisons between scenario T and scenario N. Both carbon emissions and GDP decreased as a result of carbon trading, where the significant negative effect will be aggravated as time goes by. For instance, based on Figure 4a, carbon emissions in China will decrease by around −1.26% and −3.46% in the 10th year and 20th year, respectively. The results indicate the carbon-trading mechanism is effective to reduce carbon emissions. Based on Figure 4b, the GDP in China would be reduced by −1.36% and −5.18% in the 10th year and 20th year, respectively.

**Figure 5:**
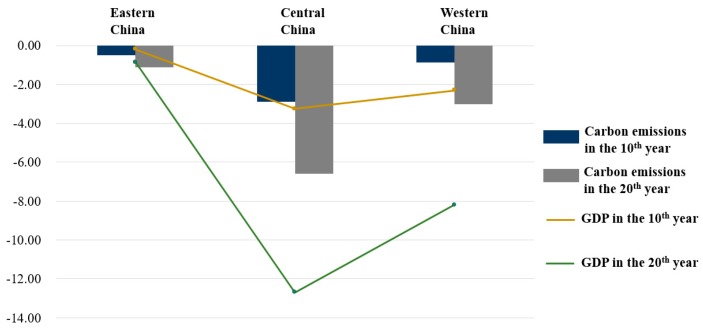
Impacts of the trading mechanism on China’s region carbon emissions and GDP under scenario T in 10th year and 20th year (%).

In particular, we probed regional development under the comparison of scenario T and scenario N, and show the difference of carbon emissions and GDP in Figure 5. As is shown in Figure 5, carbon emission reduction was −0.50% and −1.10% in 10th year and 20th year, respectively, while GDP reduction rate was −0.19% and −0.86% in 10th year and 20th year, respectively. Hence, the eastern region is least affected by a carbon-trading mechanism than other regions. Moreover, in terms of impact of a carbon-trading mechanism on both carbon emissions and GDP, the central region dominates others. For example, under scenario T, the reduction in the central region is reduced by approximately −2.89% and −6.59% in 10th year and 20th year, respectively. Then its GDP is also reduced by −3.24% and −12.69%, owing to the carbon-trading mechanism in 10th year and 20th year, respectively. As for the western region, under scenario T, the carbon emission reduction rate is −0.88% and −3.02% in 10th year and 20th year, respectively, while GDP is reduced by approximately −2.35% and −8.20% in 10th year and 20th year, respectively.

**Figure 6:**
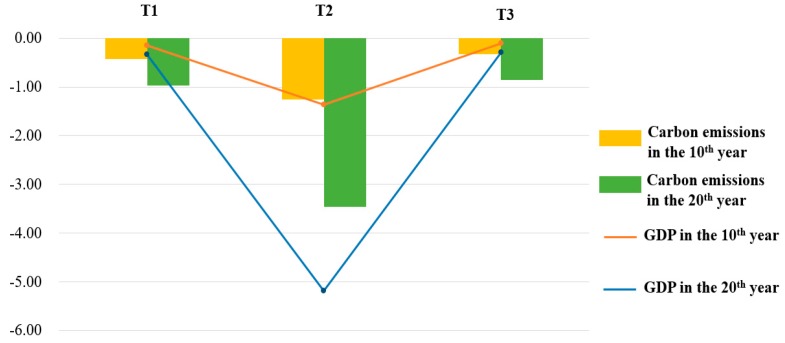
Impacts of the trading mechanism on GDP under scenario T1, T2, T3 in 10th year and 20th year (%).

**Figure 7:**
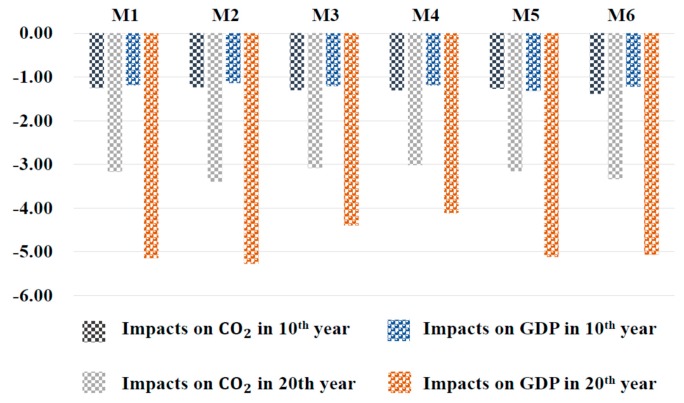
Impacts of the trading mechanism on China’s carbon emissions and GDP with different regional abatement rates under scenarios M1–M6 in 10th year and 20th year (%).

**Figure 8:**
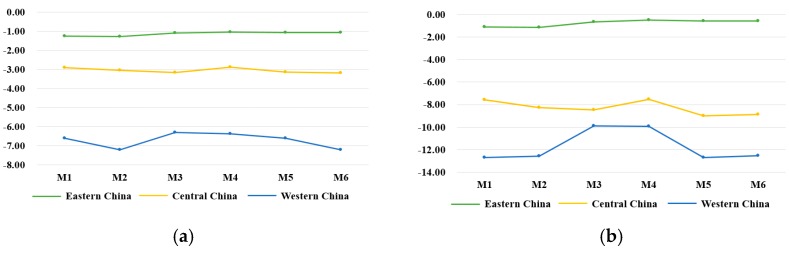
Impacts of the trading mechanism on regional carbon emissions with different regional abatement rates under scenarios M1–M6 in 20th year (%). (**a**) Regional carbon emissions, (**b**) regional GDP.

Additionally, in terms of an abatement policy, we firstly explored the abatement policy of 1.5%, 3%, and 4.5%, by Figure 6, which is a comparison among scenarios T1, T2, and T3. As is depicted in Figure 6, in the 20th year GDP is reduced by 0.33%, 5.18%, and 0.29% by scenarios T1, T2, and T3, respectively. Carbon emission is reduced by 0.97%, 3.46%, and 0.86%, respectively. Secondly, regional abatement designs were proposed to analyze how abatement rates make impact on China by scenarios M1–M6 in Figure 7. We noticed that M3 and M4 had less negative impact on both carbon emissions and GDP, which were not satisfied to our abatement goal. M2 had the most negative impact in all, which did harm to economic development. Lastly, taking account of a regional development balance, we did research on the carbon emissions and GDP of the eastern region, central region, and western region in 20th year, which is shown in Figure 8a,b, respectively. As for the western region’s development, M5 and M6 had more negative impact.

## 5. Discussion and Policy Implication

To describe a comprehensive picture for the impacts of carbon trading on China, the simulation analysis was presented by the perspective of carbon emissions, GDP, and regional development, respectively. In addition, we showed the comparisons between our work and others’.

### 5.1. Comparison with Previous Studies

In terms of methods, the multi-agents model approach that is a bottom-up simulation model was adopted by Tang et al. [16,28], focusing on the carbon market, who explored the national impact brought about by a carbon-trading market. In the studies of Tang et al. [16,28], the national carbon-trading market was modeled by 14 sectors whose period data were from the Chinese national input–output (IO) in 2007. One paper [28] analyzed a benchmark price and found 40 yuan(RMB) was better, which was used to this present study. Another paper [16] compared the mitigation rate and found that the bigger the rate was, the more reduction was. We referenced it and China’s abatement target to get the comparisons of reduction rates of 1.5%, 3%, and 4.5%. However, both papers used 2007 IO data of China as period data and just considered 14 sectors. Due to the availability of data, we adopted China’s 2012 multi-region input-output data (MRIO) and took 30 sectors into account to replace or update these data and consider more sectors, which are shown in Table 1 and Table 2, respectively. This method is also used in other countries. Based on the multi-agents approach, the carbon emissions of the transportation sector, which is responsible for 20% of Japan’s carbon emissions, have been recalculated [50]. One study used a multi-agents model to investigate the behavior of the UK energy crop market and examined the cost of emission abatement that the market might provide [51]. If period data could be collected, the method could be applied to explore many regions or counties, and from various aspects.

Furthermore, this paper contributed the extension of the multi-agents model application to the carbon-trading market from the regional perspective. Many previous studies just made analyses from a national perspective, which indicated that the requirement about mitigation was uniform to each region while ignoring the imbalance of regional economies. As for regional development on consideration of a carbon-trading market, we proposed the “discriminating policy” (M1) that the requirement for the eastern region was a bit higher, the central region basically kept unchanged, and the western region needed to be at eased, rather than the “sweeping approach” policy to various regions. Owing to differences of regions, like China’s regional energy and CO_2_ emission performance, Lin and Du suggested marketization in China’s region [52]. Liu et al. found that, due to technology inequity, such differences raise a request to revise the current energy intensity reduction targets for different provinces [53]. Additionally, Song suggested the policy focused in the western regions should build an actively guiding role by the government in carbon capture and storage (CCS) development [54]. Other flexible mechanisms, like the clean development mechanism (CDM) and joint implementation (JI), can be used to enhance cooperation of regions [55]. However, both urbanization and income growth lead to CO_2_ emissions; therefore, enforcing stricter policies should be given urgent action [56]. Therefore, adopting the “discriminating policy” is suitable for national economic development where higher abatement requirement is put on the eastern region.

Moreover, this policy (M1) has the same view with the national policy on energy conservation and emission reduction: The eastern provinces have higher energy conservation and emission reduction targets, while the western regions have lower targets due to the great ratio of its energy-consuming industries and great difficulty in completing the target.

### 5.2. General Review

During the first 10 years and more, the abatement of carbon emissions had less impact on GDP than the following years. For instance, the reduction rate of carbon emissions was approximately equal to that of GDP in 10th year, but it was growing faster after the 15th year. It is implied that China needs many years to adapt itself to the carbon-trading mechanism, due to sectors agents continuing to operate by the previous pattern, in spite of facing penalties, in the beginning of the carbon-trading mechanism. As time goes by, the reduction of carbon emissions increases and GDP decreases more.

It should be noted that carbon emissions without carbon-trading mechanism may make a worsening impact on the economy. If there is no reduction in emissions, not only the climate change could be worsened but also GDP in turn may have greater falls than those caused by the mere fact of establishing a system for regulating emissions [4,5]. In order to show the worsening effect to the economy by climate change, we used a simple numerical example to prove it. According to the scenario N, we assumed scenario N’ in which carbon emissions’ increment can bring proportional decrement (0.3) to the economy after normalizing the different unit. Based on real data (MRIO) of the base period, the difference of N’ and N can be seen and that the GDP reduced 4.97% and 44.38% in 10th year and 20th year, respectively, when both have the same carbon emissions. Obviously, the bad effect of scenario N’ on GDP is worse than scenario T. Therefore, it is necessary to implement the carbon-trading mechanism, though it brings some decrements in GDP.

From the regional perspective, the impact of the carbon-trading mechanism on the regional carbon emissions and GDP is shown in Figure 5. Two main conclusions can be drawn from the results. First, little reduction of carbon emissions can cause much reduction of GDP, except for China’s eastern region, which is less impacted by the carbon-trading mechanism. Second, the impact of the carbon-trading mechanism on the central region dominates for emission reduction, with GDP reduced much, too.

For the eastern region, its characteristics of enterprises is high-tech and knowledge-intensive with high profit. These industries themselves have low carbon emissions because of the industrial characteristics. Therefore, they may choose to face penalty and may accept to tolerate the penalty limit. 

According to the National Bureau of Statistics, the secondary industry value ratios of three regions are 48.23%, 52.05%, and 50.13%, respectively. Furthermore, secondary industrial output values include the manufacturing industry that has a huge impact on carbon emissions. Therefore, the GDP in the central region is greatly affected by carbon-trading mechanism. For the western region with industrial output values of secondary industry 50.13% in 2012, its GDP would also be cut down greatly.

Specifically, owing to the heterogeneity of a sector, these regions with characteristics of enterprises can pointedly make abatement a target by differentiating these targets across industries [51]. As for different sectors, taking T2 as an example comparing to N, we can find that (1) some sectors, like PEG increasing 13.45%, still increase carbon emissions under T2, which means these sectors are not effectively controlled by a carbon-trading mechanism, and (2) most sectors decrease emissions a lot with the economy decreasing. For example, with LCS(Leasing and commercial service), its GDP cuts 2.5 times and emissions decrease 2.2 times. Therefore, these sectors that increase carbon emission in T2, are needed to intensify the abatement requirement. However, it is also noticed that the characteristics of a sector may make it only have lower abatement potential. We can see that most sectors decreased both CO_2_ and GDP from the comparing results.

### 5.3. Discussion about National Abatement

Three scenarios T1, T2, and T3 in our design, showed differences in carbon emissions and GDP, which is shown in Figure 6. An important conclusion can be drawn that, though all scenarios will bring about a downturn in GDP and carbon emissions, the impact discriminately changed as the abatement rate changed. Furthermore, the effect of a carbon-trading mechanism on both GDP and emissions showed a U-shaped trajectory as the abatement rate increased, and the peak of the effect was reached at the abatement rate of 3%.

The main hidden reasons may be as follows: (1) The increase from 1.5% to 3% caused many small- and medium-sized enterprises to emit excessive emissions and have to face a penalty, which was a big loss for many small- and medium-sized enterprises. China has a large number of small- and medium-sized enterprises with limited scale. Moreover, it was difficult for them to be fully compensated for their increased production to cover the economic losses caused by the carbon penalty. Therefore, these enterprises chose to shrink scale to preserve economic benefits. Overall, both GDP and carbon emissions fell. (2) From 3% to 4.5%, many enterprises which were unable to effectively reduce emissions, especially large enterprises after using technology and other means, could not effectively achieve the effect of emission reduction, but the upper limit of the penalty was acceptable for them. After the maximum penalty was reached, more emissions penalties will not have to be paid. Large enterprises decided to increase production, which meant their purpose was not only to make up for the economic losses caused by penalties, but also to effectively expand the scale of production and increase revenues. Therefore, from T3 to T2, the carbon emissions increased, and the benefits increased, too. But for small- and medium-sized enterprises, the phenomenon of downsizing existed still. Consequently, the abatement rate in China’s carbon-trading market should be carefully set to be neither too low nor too high.

### 5.4. Discussion about Regional Abatement

For these regional abatement policies, the impact of a trading mechanism on China’s emissions and GDP are shown in Figure 7.

From scenarios M3 and M4, we can see those scenarios had lower reduction both of carbon emissions and GDP. Owing to the abatement rate of 4.5% was more than scenario T, the likely reason probably was that the higher rate makes the central region fit the mechanism in advance, or the easily taxing penalty led most sectors agents in the central region to be more willing to keep initial development and production regardless of penalty, with a result of less carbon reduction than with other regional policies. All in all, this measure goes against what we expect it will be. So, these two scenarios should be abandoned, because making much progress to decrease emissions is our pursuit, and these two policies work worse than the abatement of other scenarios with results of carbon reduction −3.08%, −3.02% in the 20th year, respectively. Moreover, carbon emissions in scenario M2 was the lowest among the six abatement policies with −3.40%, but decreased most in GDP with −5.28% in the 20th year, which indicates it satisfied the demand on abatement with the price of damaging the economy.

As regards M1, M5, and M6, there are some expansions in detail from a regional perspective to identify which policy is more appropriate for China’s regional development. For convenience and clearly showing the difference among regions, comparisons of carbon emissions and GDP in the 20th year are illustrated in Figure 8.

It is a remarkable attention that GDP of the western region was supposed to be caressed by policy. Through comparison, the decrement of GDP in the western region was −7.56%, −8.98%, and −8.89% in scenarios M1, M5, and M6, respectively, where M5 and M6 were of more harm to the western economic development. To have a deep interest in the economic development of western China, scenario M1 was adopted to avoid economic imbalances in the regions. In conclusion, although the scenario of M1 in the 20th year was neither the biggest reduction in GDP nor the least carbon reduction, this policy benefitted regional development as a whole, which avoided unbalanced development. That is to say, M1 assigned different requirements towards regions according to their different characteristics. Explicitly speaking, the requirement for the eastern region was a bit higher, the central region basically remained unchanged, and the western region needed to put the abatement requirement in a more open, comfortable zone. This policy with a discriminating requirement to different regions is not a “sweeping approach”.

### 5.5. Policy Implications

The above simulation results can be of help to China’s carbon trading mechanism, which can be summarized as follows. Generally, a carbon-trading mechanism, an effective tool, reduces total carbon emissions with a relatively small negative impact on GDP. In other words, the emergence of a carbon-trading mechanism keeps carbon emissions reduced with time, and, furthermore, makes significant negative impact on GDP, which is inevitable in this policy performance. Besides, too low or too high of the abatement rate is unfit because it does not satisfy abatement expectations or targets, which can be seen from comparisons of scenarios T1, T2, and T3. Therefore, a reasonable formulation of the abatement rate is the premise of both economic and emission reduction in a carbon-trading mechanism that is an actually effective policy tool. In addition, China, the largest carbon emission country, actively take its responsibility, though there are many obstacles and difficulties in pushing the progress of low carbon transition.

Moreover, the different reduction rates for the three regions can bring various impacts for regional development. From the regional policies, we can see that abandoning a “sweeping approach” is beneficial to not only the western region but also to national development through comparing scenarios M1–M6. Therefore, we can get some policy implications as follows:

(1) The proportion of the secondary industry in the central region is large, and the number of enterprises are in bulk. Through scenario T, it was obvious to see that the central region was most affected by the carbon market.
(2) The eastern region was less affected by the emission reduction policies than other regions, so the emission reduction can be slightly increased. From M1–M6, it was difficult for the eastern region, the GDP leader, to reduce the emissions, and the fine limit was not relatively high. Therefore, most enterprises will not cut production and not meet the emission reduction requirements, because the worst condition is facing a penalty in an acceptable range. In other words, the eastern region is more economically developed than the western region. In order to accommodate regional development, it is reasonable for the eastern region to bear the cost of emissions reduction required by some countries for the western region. Therefore, it is sensible to increase the emission reduction requirements of the eastern region.
(3) The government needs to seriously take a look at the western region’s development to avoid deepening the cap of regional imbalance. Like the scenario M1, the policy decreased the requirement to the west. Only in this way, can regional development steadily make progress.

The “discriminating policy” can have a good effect on public health in the future, which can be showed in three aspects: (1) Through balancing GDP and carbon emissions by different regional abatement requirements, people in each region can enjoy a better life not only in their living environment but also in economic activities. (2) Implementing a carbon trading mechanism can reduce carbon emissions, which is effective to defend people against diseases of environmental pollution. It is acknowledged that air pollution makes much effect on health, through inhalation, skin contact, and eye contact ingestion, resulting in carcinogenic effects through long-term exposure [57]. M1 reduced carbon emissions more than 3% from simulation to the 20th year. (3) Although the economy decreased under the related policy, it was still better than scenario N’ that did not have a carbon emissions regulation. For improving environment, the little loss of economy can be tolerated, like the “discriminating policy”, with reasonable economic loss, which stabilized the economy to make sustainable development practical.

It is noticed that some important aspects can have much effect on making policy but we ignore them. (1) For production system improvements, there are many aspects showing alternatives to reduce emissions. One clear example would be to invest in R&D&I(Research & Development & Investment) to improve production systems towards clean energy and a more efficient processes. Another is improving the technology level. With the development of technology, as to firms, the carbon emissions with productivity are reduced. The long-term benefits that emission-saving technologies could make impact on GDP can be seen in previous studies. Therefore, encouraging firms to make technology improvements could be another policy orientation. (2) For the economic environment, the multi-agents model simulates long-term process, but the different economic environment that happens during that time could affect economic decisions of agents in an emission-trading mechanism. In the long period, the role of economic variables in determining the convenience of an investment in energy efficiency could dramatically change [58]. Therefore, the policies face uncertainties, which means the “discriminating policy” may not suitable for the long term. (3) For valid alternative mechanisms in the carbon abatement process, firms also have other alternatives to reduce their emissions, beyond buying or selling emission rights, like a clean development mechanism (CDM) and joint implementation (JI). (4) For carbon transfer, there is an obvious phenomenon that many developed countries could possibly relocate their industries to third countries, expecting to reduce their carbon emissions but with emissions increases in others, like China. Therefore, in some carbon quota calculations mistakes may exist, owing to complexity of actual accounting.

## 6. Conclusions

This paper proposes a China carbon-trading model by multi-agents technology, in which sector agents and government agents are considered. The carbon-trading mechanism keeps carbon emissions reduced with time. Furthermore, it makes a significant negative impact on GDP, which is inevitable during performance. In particular, regional abatement policies presented in some scenarios demonstrated a discriminating requirement to different regions, which is beneficial to China’s regional development rather than a “sweeping approach”. The abatement requirement for the western region needs to be at eased, but the eastern region is opposite.

Obviously, a carbon-trading mechanism can effectively reduce carbon emissions and make a negative impact on GDP. The resulted from comparing T1, T2, and T3, and further indicates that the abatement rate in China’s carbon-trading market should be carefully set to be neither too low nor too high. However, if making the abatement requirements clear and definite in a regional level according to the characteristics of a sector is needed, it is important to use different requirements again.

When comparing regional abatement policies, national abatement can neither be too high nor too low for reducing carbon emissions and maintaining economic stability. Moreover, the “sweeping approach” policy should be replaced by a regional “discriminating policy”, that the abatement requirement to the western region is lower and to the eastern region is relatively higher, which is more beneficial to China’s regional development. Explicitly, the requirement for the eastern region is a bit higher, the central region basically remains unchanged, and the western region needs to be in a more open, comfortable zone.

However, there are some limitations in our works. This method has uncertainties in the long-term simulation process. It is important to note that each subject’s interactions tend to produce different results, which is the limitation of the multi-agents model. Some extensions can be considered in future work. To understand the extent to which the value of each parameter affects the results could be one direction. In other words, the extensive sensitivity analysis can be conducted in our future work. Additionally, some other ways to achieve abatement can be jointly considered, like clean development mechanism (CDM).

## Figures and Tables

**Table 1:** Codes for sectors.

ID	Code	Full Title of the Sector
Sector 1	AGR	Agriculture
Sector 2	COM	Coal mining
Sector 3	PEG	Petroleum and gas
Sector 4	MEM	Metal mining
Sector 5	NOM	Nonmetal mining
Sector 6	FPT	Food processing and tobaccos
Sector 7	TEX	Textile
Sector 8	CLF	Clothing, leather, fur, etc.
Sector 9	WPF	Wood processing and furnishing
Sector 10	PPS	Paper making, printing, stationery, etc.
Sector 11	PRC	Petroleum refining, coking, etc.
Sector 12	CHI	Chemical industry
Sector 13	NOP	Nonmetal products
Sector 14	MET	Metallurgy
Sector 15	MEP	Metal products
Sector 16	GSM	General and specialist machinery
Sector 17	TRE	Transport equipment
Sector 18	ELE	Electrical equipment
Sector 19	ECE	Electronic equipment
Sector 20	INE	Instrument and meter
Sector 21	OTM	Other manufacturing
Sector 22	EHP	Electricity and hot water production and supply
Sector 23	GWP	Gas and water production and supply
Sector 24	CON	Construction
Sector 25	TRS	Transport and storage
Sector 26	WHR	Wholesale and retailing
Sector 27	HOR	Hotel and restaurant
Sector 28	LCS	Leasing and commercial service
Sector 29	SCR	Scientific research
Sector 30	OTS	Other service

**Table 2:** Codes for provinces.

ID	Code	Region	Province
Province 1	BJ	Eastern region	Beijing
Province 2	TJ	Eastern region	Tianjin
Province 3	HB1	Eastern region	Hebei
Province 4	SX1	Central region	Shanxi
Province 5	IM	Western region	Inner Mongolia
Province 6	LN	Eastern region	Liaoning
Province 7	JL	Central region	Jilin
Province 8	HL	Central region	Heilongjiang
Province 9	SH	Eastern region	Shanghai
Province 10	JS	Eastern region	Jiangsu
Province 11	ZJ	Eastern region	Zhejiang
Province 12	AH	Central region	Anhui
Province 13	FJ	Eastern region	Fujian
Province 14	JX	Central region	Jiangxi
Province 15	SD	Eastern region	Shandong
Province 16	HN1	Central region	Henan
Province 17	HB2	Central region	Hubei
Province 18	HN2	Central region	Hunan
Province 19	GD	Eastern region	Guangdong
Province 20	GX	Western region	Guangxi
Province 21	HN3	Eastern region	Hainan
Province 22	CQ	Western region	Chongqing
Province 23	SC	Western region	Sichuan
Province 24	GZ	Western region	Guizhou
Province 25	YN	Western region	Yunnan
Province 26	SX2	Western region	Shaanxi
Province 27	GS	Western region	Gansu
Province 28	QH	Western region	Qinghai
Province 29	NX	Western region	Ningxia
Province 30	XJ	Western region	Xinjiang

**Table 3:** Scenarios of multi-region policy design.

Policy	Scenario	Region	Reduction Rate (%)
No Trading	N	-	-
No Trading under climate effect	N’	-	-
Typical	T	-	3
Abatement policy	T1	-	1.5
	T2	-	3
	T3	-	4.5
	M1	Eastern	4.5
		Central	3
		Western	1.5
	M2	Eastern	4.5
		Central	1.5
		Western	3
	M3	Eastern	1.5
		Central	4.5
		Western	3
Regional policy	M4	Eastern	3
		Central	4.5
		Western	1.5
	M5	Eastern	1.5
		Central	3
		Western	4.5
	M6	Eastern	3
		Central	1.5
		Western	4.5

**Table 4:** Simulation errors for provincial GDP and carbon emissions under scenario N in the base year (%).

	**BJ**	**TJ**	**HB1**	**LN**	**SH**	**JS**	**ZJ**	**FJ**	**SD**	**GD**
GDP	−1.30	−1.66	−1.73	−1.73	0.63	−1.35	−1.72	−1.44	−1.32	9.02
CO_2_	0.71	6.36	−1.10	1.32	−2.91	−3.01	−4.87	4.22	−1.63	3.98
	**HN1**	**SX1**	**IM**	**JL**	**HL**	**AH**	**JX**	**HN2**	**HB2**	**HN3**
GDP	1.97	−1.72	−1.73	−1.73	−1.70	−3.04	−1.00	−1.73	−4.50	−1.70
CO_2_	7.87	6.88	−2.28	2.91	0.25	−3.47	−9.00	−2.21	0.78	−1.95
	**GX**	**CQ**	**SC**	**GZ**	**YN**	**SX2**	**GS**	**QH**	**NX**	**XJ**
GDP	−1.71	−2.16	−1.64	−1.01	−1.15	−1.71	−0.16	−1.23	−0.87	−2.00
CO_2_	−2.95	−9.67	6.54	4.81	5.97	8.26	7.73	6.75	−8.00	8.92
